# Chemerin acts via CMKLR1 and GPR1 to stimulate migration and invasion of gastric cancer cells: putative role of decreased TIMP-1 and TIMP-2

**DOI:** 10.18632/oncotarget.26414

**Published:** 2019-01-04

**Authors:** J. Dinesh Kumar, Iman Aolymat, Laszlo Tiszlavicz, Zita Reisz, Hanan M. Garalla, Rob Beynon, Deborah Simpson, Graham J. Dockray, Andrea Varro

**Affiliations:** ^1^ Department of Cellular and Molecular Physiology, Institute of Translational Medicine, University of Liverpool, Liverpool, UK; ^2^ Department of Pathology, University of Szeged, Szeged, Hungary; ^3^ Centre for Proteome Research, Institute of Integrative Biology, University of Liverpool, Liverpool, UK

**Keywords:** chemerin, gastric cancer, myofibroblasts, proteomic

## Abstract

The chemokine-like peptide, chemerin, stimulates chemotaxis in several cell types. In this study we examined the expression of putative chemerin receptors in gastric cancer and the action of chemerin on cancer cell migration and invasion. Immunohistochemical studies of gastric tumors identified expression of two putative receptors, chemokine-like receptor-1 (CMKLR1) and G-protein coupled receptor 1(GPR1), in cancer cells; there was also some expression in stromal myofibroblasts although generally at a lower intensity. The expression of both receptors was detected in a gastric cancer cell line, AGS; chemerin itself was expressed in cultured gastric cancer myofibroblasts but not AGS cells. Chemerin stimulated (a) morphological transformation of AGS cells characterized by extension of processes and cell scattering, (b) migration in scratch wound assays and (c) both migration and invasion in Boyden chamber chemotaxis assays. These responses were inhibited by two putative receptor antagonists CCX832 and α-NETA. Inhibition of receptor expression by siRNA selectively reduced CMKLR1 or GPR1 and inhibited the action of chemerin indicating that both receptors contributed to the functional response. Using a proteomic approach employing stable isotope dynamic labeling of secretomes (SIDLS) to selectively label secreted proteins, we identified down regulation of tissue inhibitors of metalloproteinease (TIMP)1 and TIMP2 in media in response to chemerin. When cells were treated with chemerin and TIMP1 or TIMP2 the migration response to chemerin was reduced. The data suggest a role for chemerin in promoting the invasion of gastric cancer cells via CMKLR1 and GPR1at least partly by reducing TIMP1 and TIMP2 expression. Chemerin receptor antagonists have potential in inhibiting gastric cancer progression.

## INTRODUCTION

Gastric cancer is considered to be the second commonest cause of cancer mortality worldwide [1]. Early detection favours survival but nevertheless the prognosis is dismal for most patients with 5 year survival in many parts of the world of about 20% [2]. It is well established that infection with *Helicobacter pylori* carries an increased risk of gastric cancer but progression occurs over many decades following a well document sequence of chronic inflammation, atrophy, metaplasia and dysplasia [3, 4]. While genetic, dietary and environmental factors may all play a role in those patients who do progress to cancer, the mechanisms promoting tumor invasion and metastasis remain incompletely understood.

It is now well recognised that in solid tumors there are interplays between cancer cells and stromal cells that strongly influence the disease process [5]. In particular, cancer cell growth depends on the appropriate microenvironment which in turn is determined by non-neoplastic stromal cells. There are important roles for immune and angiogenic cells [6]; but in addition cells of fibroblastic lineages are now seen as key contributors to the tumor microenvironment [7]. Functional differences between normal and cancer-associated fibroblasts are recognised to underpin the role of the latter in promoting tumor growth. Myofibroblasts are an important subset of fibroblasts and differences in gene expression, protein secretion, miRNA profiles, DNA methylation, cell proliferation and motility have all been described for cancer-associated myofibroblasts (CAMs) compared with normal tissue myofibroblasts or cancer adjacent tissue myofibroblasts [8–11]. In the case of squamous esophageal cancer, the chemokine-like peptide chemerin has recently been described as upregulated in CAMs and to stimulate esophageal cancer cell invasion [12, 13].

Chemerin (also known as tazarotene induced gene 2, TIG2; retinoic acid receptor responder 2, RARRES2) is an 18kDa protein, which is cleaved in the C-terminal region to generate an active product [14]. It is quite widely expressed in liver, placenta and adipocytes. Two putative functional receptors have been identified: CMKLR1 (also known as ChemR23, TIG2 receptor) and GPR1 [14–17]; chemokine receptor-like 2 (CCRL2) may also bind chemerin and aid in its presentation to CMKLR1 [18, 19]. There have been reports that chemerin is increased in blood in gastric cancer patients [20]. Moreover, the extensively used gastric cancer cell line, AGS, has been reported to express chemerin receptors and respond to chemerin by increased migration [20, 21]. However, the expression of receptors in primary gastric cancers is largely unexplored and understanding of the mechanism of action of chemerin in this condition is still at an early stage. We now report that both CMKLR1 and GPR1 are expressed in gastric cancer and in AGS cells, and both mediate migratory and invasive responses. Interestingly, a proteomic study identified down-regulation of tissue inhibitors of metalloproteinases (TIMPs) as potentially implicated in the migratory response.

## RESULTS

### Expression of chemerin receptors in gastric cancer

**Figure 1:**
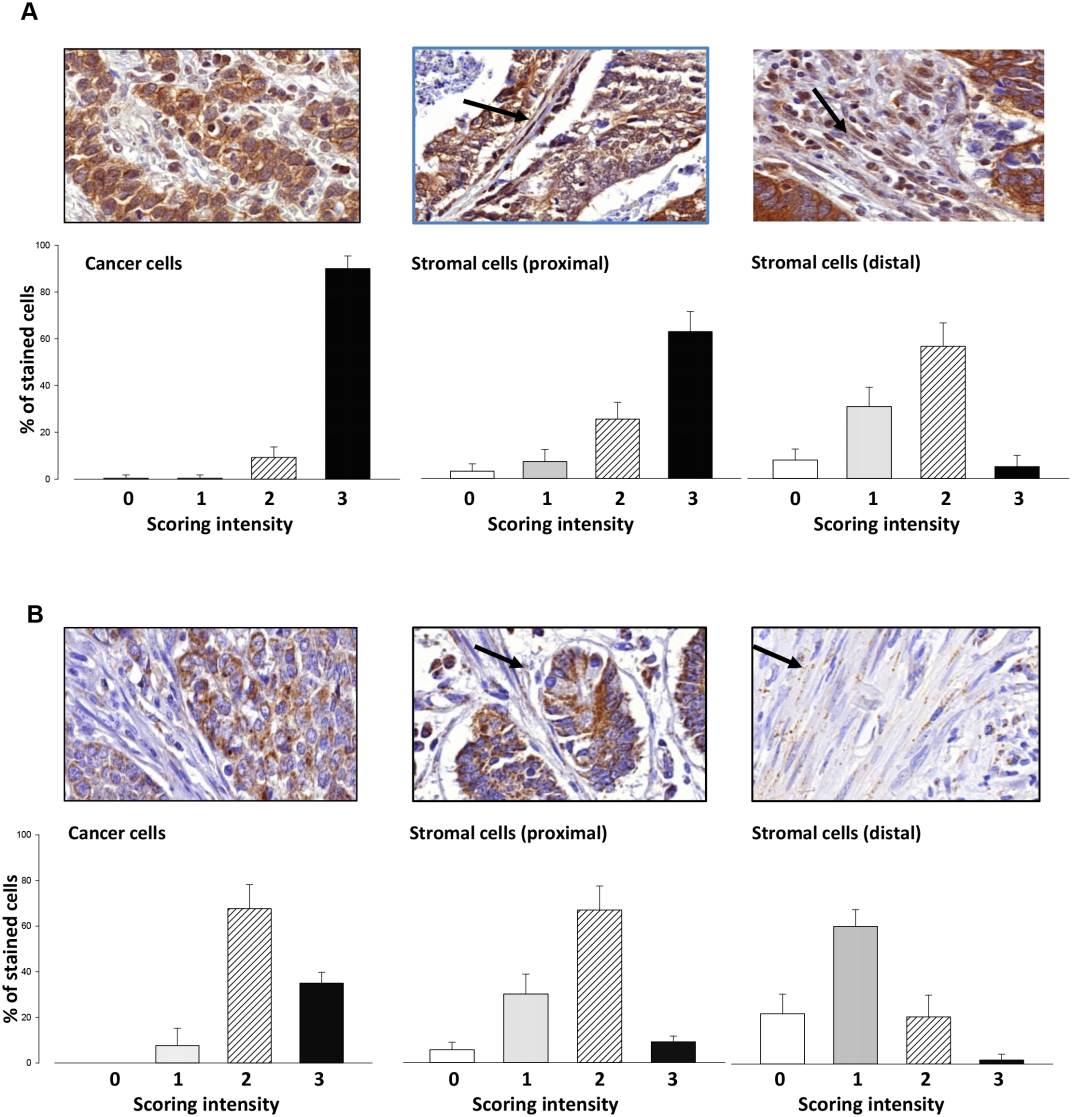
Expression of CMKLR1 and GPR1 in gastric cancer **(A)** Immunohistochemical localization of CMKLR1 in gastric tumors (top panels) and quantification in lower panels based on the proportion of cells in each compartment scored 0 – 3 for intensity; left, cancer cells; centre, stromal cells close to tumor cells (proximal); right, stromal cells distal to tumor cells (distal). **(B)** Similar data for GPR1. Bar graphs show data for 15 patients, mean ± S.E.

Immunohistochemical studies on 15 patients with gastric cancer revealed CMKLR1 at high intensity in virtually all cancer cells (Figure 1A) with no obvious differences between intestinal, diffuse or mixed gastric cancers, or TNM stage. There was also expression in spindle-shaped stromal cells, consistent with a myofibroblast phenotype; the intensity of staining of stromal cells was greatest in those cells adjacent to tumor cells (Figure 1A, centre panel) compared with those that were distant (Figure 1A, right). There was also expression of GPR1 in most cancer cells although the intensity of staining was lower than CMKLR1; moreover, GPR1 was also expressed in myofibroblasts particularly those adjacent to tumor cells (Figure 1B).

### Chemerin mediates myofibroblast chemotactic effects on cancer cells

**Figure 2:**
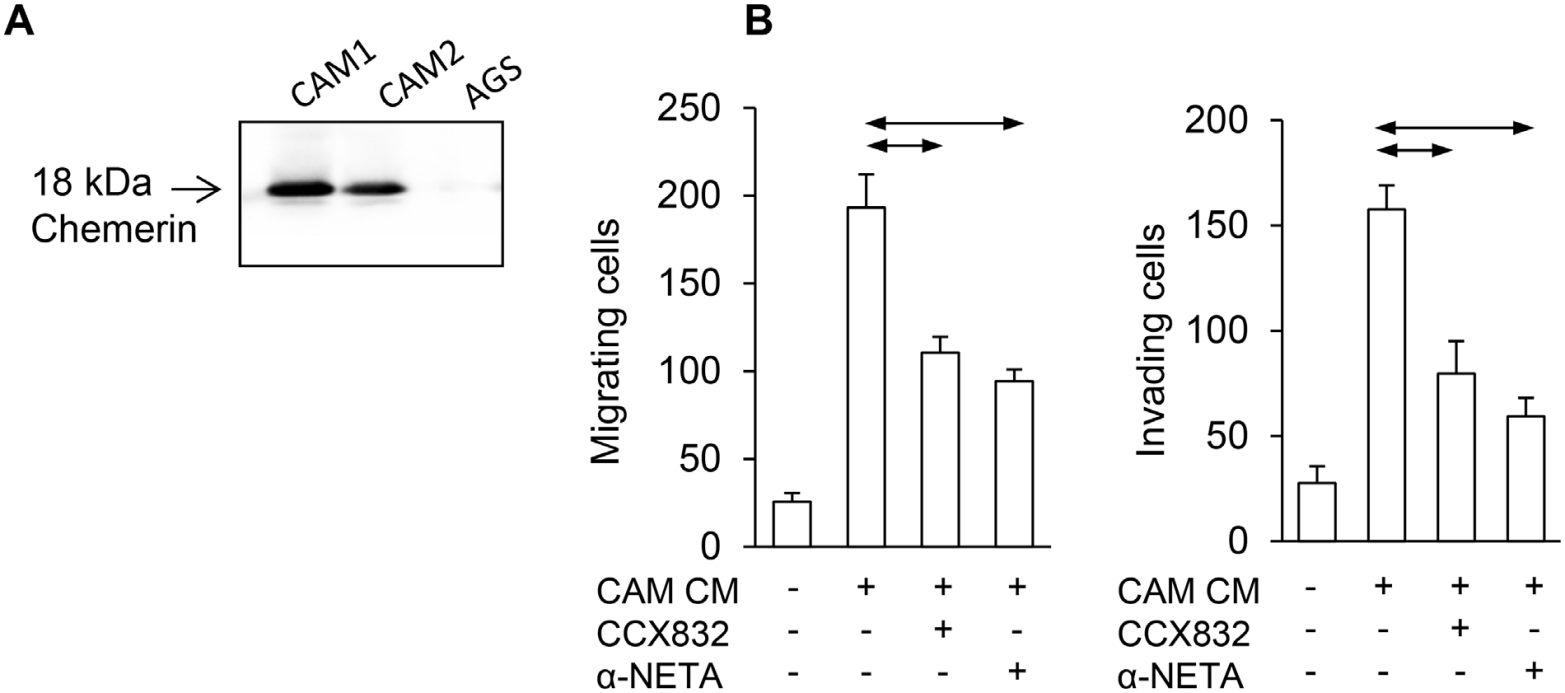
Chemerin mediates myofibroblast effects on AGS cancer cells **(A)** Western blot shows expression of chemerin in two different gastric cancer-associated myofibroblasts (CAM1 and CAM2) but not AGS cells. **(B)**, Left, conditioned medium from gastric CAMs (CAM CM) stimulates migration of AGS cells in Boyden chamber chemotaxis assays and the response is inhibited by CCX832 (1μM) and α-NETA (5μM); right, similar results for Boyden chamber invasion assays (n=3). Horizontal lines, p<0.05, ANOVA.

To establish whether chemerin is a potential mediator of stromal-cancer cell interactions in gastric cancer, as in esophageal squamous cancer [13], we sought evidence by western blot for chemerin expression in gastric myofibroblasts: two different gastric CAMs secreted chemerin into the medium while the cancer cell line, AGS, did not (Figure 2A). Evidence that CAMs secrete chemerin in functionally relevant concentrations was indicated by the observation that conditioned medium from gastric CAMs stimulated AGS cell migration in Boyden chamber experiments, and the effects were inhibited by two chemerin receptor antagonists, CCX832 and α-NETA (Figure 2B).

### Chemerin stimulates morphological transformation of AGS cells

**Figure 3:**
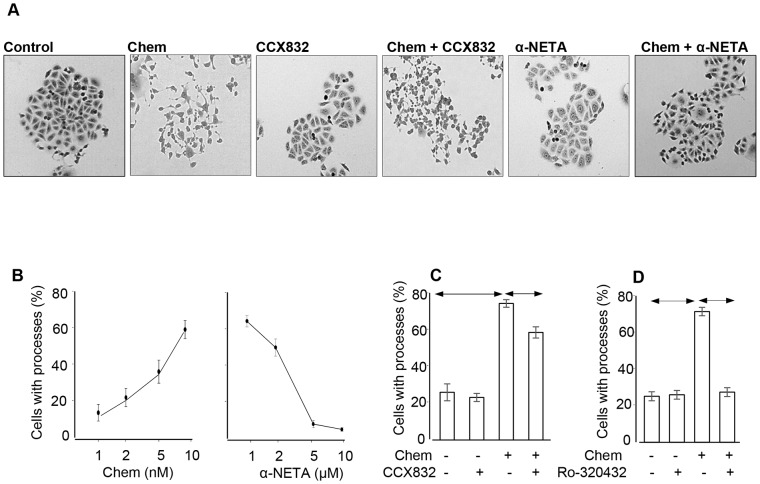
Chemerin induces a morphological transformation of AGS cells **(A)** representative images of sub-confluent AGS cells showing that chemerin (Chem, 10 nM) induces cell scattering and extension of processes which is reduced by addition of CCX832 (1μM) and α-NETA (5μM); the latter have no effect alone. **(B)** Dose-response relationships for the action of chemerin alone in stimulating process extension by AGS cells (left) and of graded concentrations of α-NETA in inhibiting the effects of 10 nM chemerin (right). **(C)** Quantitative assessment of the proportion of cells exhibiting extension of processes after treatment with chemerin (10 nM), CCX832 (1μM) and Ro-320432 (1 μM); (n=3). Horizontal lines, p<0.05, ANOVA.

To directly examine the effects of chemerin on gastric cancer cells, we then treated sub-confluent AGS cells with chemerin. Over a period of 7 h chemerin-treated AGS cells exhibited a morphological transformation characterized by extension of processes and cell scattering (Figure 3A). Quantification of transformed cells revealed a concentration-dependent response that was almost completely inhibited by α-NETA at 5 μM, and was significantly inhibited by CCX832 at 1μM (Figure 3B and 3C). There is a similar transformation, which has been characterised as epithelial-mesenchymal transition (EMT), in response to gastrin which is protein kinase C (PKC) mediated [22]; in the present study inhibition of PKC using Ro-320432 also abolished the transformation in response to chemerin (Figure 3D).

### Chemerin stimulates AGS cell migration and invasion

**Figure 4:**
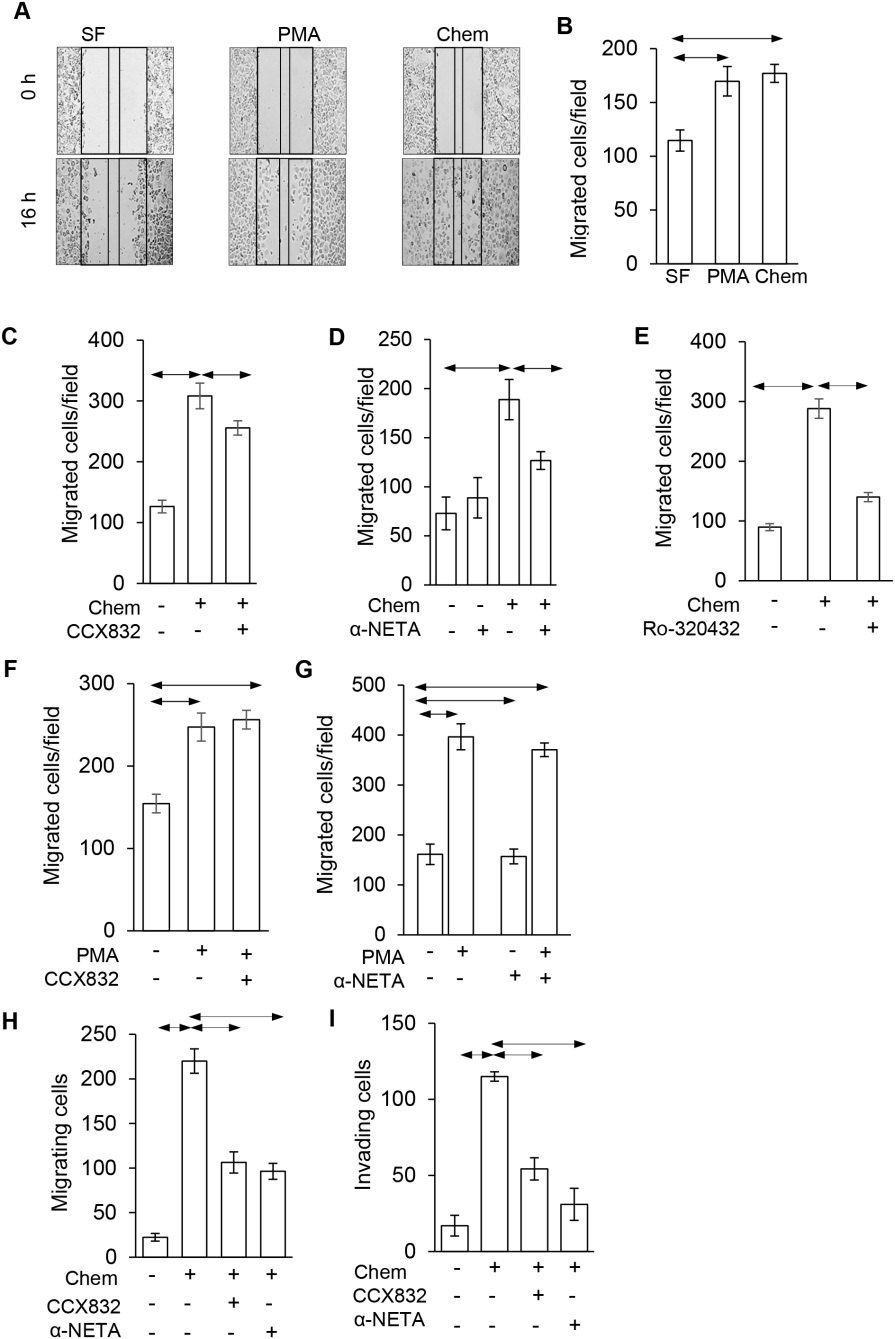
Chemerin stimulated AGS cell migration is inhibited by CCX832 and α-NETA **(A)** Representative images showing scratch wound assays at 0 and 16 h and effect of chemerin (Chem, 10 nM) and phorbol 12-myristate 13-acetate (PMA, 100 nM) compared with control, serum-free (SF) medium. **(B)** Quantification of migrating cells in a defined area in scratch wound assays in response to chemerin and PMA. **(C)** The action of chemerin in scratch wound assays is inhibited by CCX832 (1 μ), **(D)** by α-NETA (5 μM) and **(E)**, by Ro-320432 (1μM). **(F)**, The action of PMA in scratch wound assays is not inhibited by CCX832, or **(G)** α-NETA. **(H)**, In Boyden chamber chemotaxis migration assays, the effect of chemerin is inhibited by CCX823 and α-NETA; **(I)** similar data for Boyden chamber invasion assays. Horizontal lines, p<0.05 ANOVA.

In order to examine the migratory response to chemerin in more detail we first used scratch wound assays (Figure 4A). These showed increased migration in response to both chemerin and phorbol 12-myristate 13-acetate (PMA) (Figure 4B); the effect of chemerin was inhibited by 1 μM CCX832 (Figure 4C), 5 μM α-NETA (Figure 4D) and 1 μM Ro-320432 (Figure 4E). The specificity of action of CCX832 and α-NETA was indicated by the fact that increased migration in response to PMA was not inhibited CCX832 or α-NETA (Figure 4F and 4G). Similarly, in Boyden chamber chemotaxis migration (Figure 4H) and invasion (Figure 4I) assays there was also a strong response to chemerin that was significantly inhibited by CCX832 and α-NETA.

### Both CMKLR1 and GPR1 mediate the effect of chemerin

**Figure 5:**
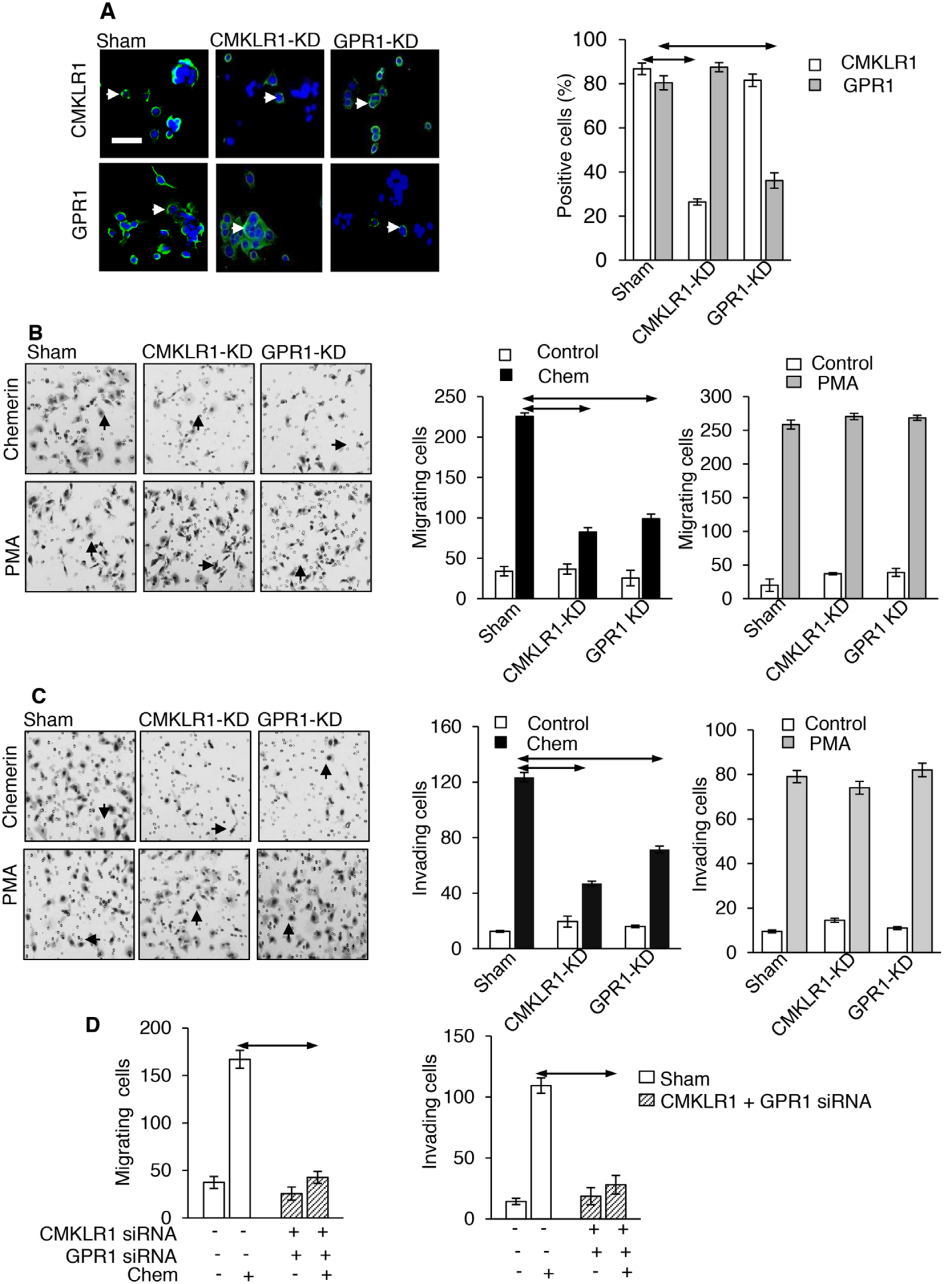
CMKLR1 and GPR1 mediate the effects of chemerin on AGS cells **(A)** Left, Immunocytochemical localization of CMKLR1 and GPR1 on AGS cells and selective knockdown (CMKLR1-KD and GPR1-KD, respectively) by siRNA treatment (arrows, positively stained cells); right, quantification of positively stained cells after siRNA knockdown (open bars, proportion of cells exhibiting CMKLR1 staining; shaded bars, proportion of cells exhibiting GPR1 staining). **(B)** Left, representative images from Boyden chamber migration experiments after CMKLR1 and GPR1 knockdown (arrows, migrating cells); centre, quantification of migration responses to chemerin (Chem, 10 nM) after receptor knockdown; right, quantification of migration responses to PMA (100 nM) after receptor knockdown. **(C)**, Left, representative images from Boyden chamber invasion experiments after CMKLR1 and GPR1 knockdown (arrows, invading cells); centre, 10 quantification of invasion responses to 10 nM chemerin after receptor knockdown; right, quantification of invasion responses to 100 nM PMA after receptor knockdown. **(D)**, Left, Boyden chamber migration responses after double knockdown of CMKLR1 and GPR1; right Boyden chamber invasion responses after double knockdown of CMKLR1 and GPR1. Horizontal arrows, p<0.05, ANOVA.

We then examined the role of the two putative receptors by their selective knockdown in AGS cells. Immunohistochemistry identified both CMKLR1 and GPR1 in virtually all AGS cells (Figure 5A). Treatment of cells with siRNA for CMKLR1 reduced the number of cells expressing the receptor by approximately 75%, but did not change the proportion of cells expressing GPR1; conversely, siRNA inhibition of GPR1 reduced the proportion of cells expressing the receptor by approximately 60% but did not change the expression of CMKLR1 (Figure 5A). In Boyden chamber migration assays, selective knockdown of CMKLR1 and GPR1 significantly inhibited the response to chemerin, although in each case it was not completely suppressed (Figure 5B). The response to PMA which was run as a positive control was not influenced by receptor knockdown (Figure 5B). Similarly, in invasion assays, siRNA knockdown of both receptors inhibited but did not abolish the response to chemerin, while that to PMA was unaffected (Figure 5C). When siRNA knockdown of both receptors was performed simultaneously there was virtually complete inhibition of both migration and invasion responses (Figure 5D).

### Stable isotope dynamic labeling of secretomes (SIDLS) identification of secreted proteins in chemerin-treated AGS cells

We then employed a proteomic approach (SIDLS) to identify changes in secretome proteins that were potentially relevant to the actions of chemerin described above. Proteomic studies of cell media frequently identify both authentic secretory proteins and intracellular proteins released by cell damage; because secretory proteins turnover more rapidly than intracellular proteins a shortened labeling period preferentially labels secretory proteins [23, 24]. Thus a total of 1759 proteins were identified of which 240 had a labeling (heavy to light amino acid) ratio >0.01 indicating a degree of preferential labeling. As an independent check on the identification of secretory proteins, we then filtered the secretome list by searching for classical secretory proteins defined on the basis of a SignalP score of >0.5 Of the latter there were 36 that had been labelled in 2 or more tryptic peptides and interestingly the H/L ratios were typically <0.5 in these proteins (Table 1) indicating inhibition of expression by chemerin.

**Table 1:** Secreted proteins with a differential incorporation of heavy and light isotope. Proteins were filtered on the basis of H/L ratio >0.01 and SigP score >0.5. The Table lists name, sub-cellular localisation and H/L ratio

No.	Description (Human Proteins)	Accession	Subcellular Localisation	H:L Ratio Chemerin: Control
1	Insulin-like growth factor-binding protein 6 (IBP6)	P24592	Secreted	0.35
2	Cathepsin D (CATD)	P07339	Secreted	0.314
3	Prosaposin (SAP)	P07602	Secreted	0.278
4	Calsyntenin-1 (CSTN1)	O94985	Endoplasmic reticulum membrane	0.267
5	Sulfhydryl oxidase 1 (QSOX1)	O00391	Secreted	0.256
6	Pro-MCH OS=Homo sapiens (PMCH)	P20382	Secreted	0.252
7	Serine protease 23 (PRS23)	O95084	Secreted	0.249
8	Metalloproteinase inhibitor 1 (TIMP1)	P01033	Secreted	0.229
9	Thrombospondin-1 (TSP1)	P07996	Endoplasmic reticulum	0.211
10	Cystatin-C (CYTC)	P01034	Secreted	0.21
11	Metalloproteinase inhibitor 2 (TIMP2)	P16035	Secreted	0.21
12	Kallikrein-6 (KLK6)	Q92876	Secreted	0.209
13	Amyloid beta A4 protein (APP)	P05067	Membrane	0.207
14	Trypsin-1 ([TRY1)	P07477	Secreted	0.183
15	Cystatin-SN (CYTN)	P01037	Secreted	0.181
16	Basement membrane-specific heparan sulfate proteoglycan core protein (PGBM)	P98160	Secreted	0.174
17	Agrin (AGRN)	O00468	Secreted	0.167
18	Zinc transporter ZIP10 (S39AA)	Q9ULF5	Membrane	0.153
19	Growth/differentiation factor 15 (GDF15)	Q99988	Secreted	0.15
20	Amyloid-like protein 2 (APLP2)	Q06481	Membrane	0.141
21	Dickkopf-related protein 1 (DKK1)	O94907	Secreted	0.139
22	Peptidyl-glycine alpha-amidating monooxygenase (AMD)	P19021	Membrane/Secreted	0.129
23	Beta-2-microglobulin (B2MG)	P61769	Secreted	0.127
24	Dystroglycan (DAG1)	Q14118	Secreted	0.122
25	Trypsin-2 (TRY2)	P07478	Secreted	0.121
26	Kunitz-type protease inhibitor 1 (SPIT1)	O43278	Secreted.	0.108
27	HLA class I histocompatibility antigen, A-2 alpha chain (HLA-A)	P01892	Membrane	0.105
28	Dipeptidyl peptidase 1 (CATC)	P53634	Lysosome	0.094
29	Disintegrin and metalloproteinase domain-containing protein 9 (ADAM9)	Q13443	Secreted	0.074
30	Protein disulfide-isomerase A3 (PDIA3)	P30101	Endoplasmic reticulum	0.073
31	Growth-regulated alpha protein (CXCL1)	P09341	Secreted	0.067
32	Midkine (MDK)	P21741	Secreted	0.063
33	Interleukin-8 OS=Homo sapiens (CXCL8)	P10145	Secreted	0.05
34	Protein disulfide-isomerase (P4HB)	P07237	Endoplasmic reticulum	0.047
35	Nucleobindin-1 OS=Homo sapiens (NUCB1)	Q02818	Secreted	0.047
36	Calreticulin OS=Homo sapiens (CALR)	P27797	Secreted	0.039

**Figure 6:**
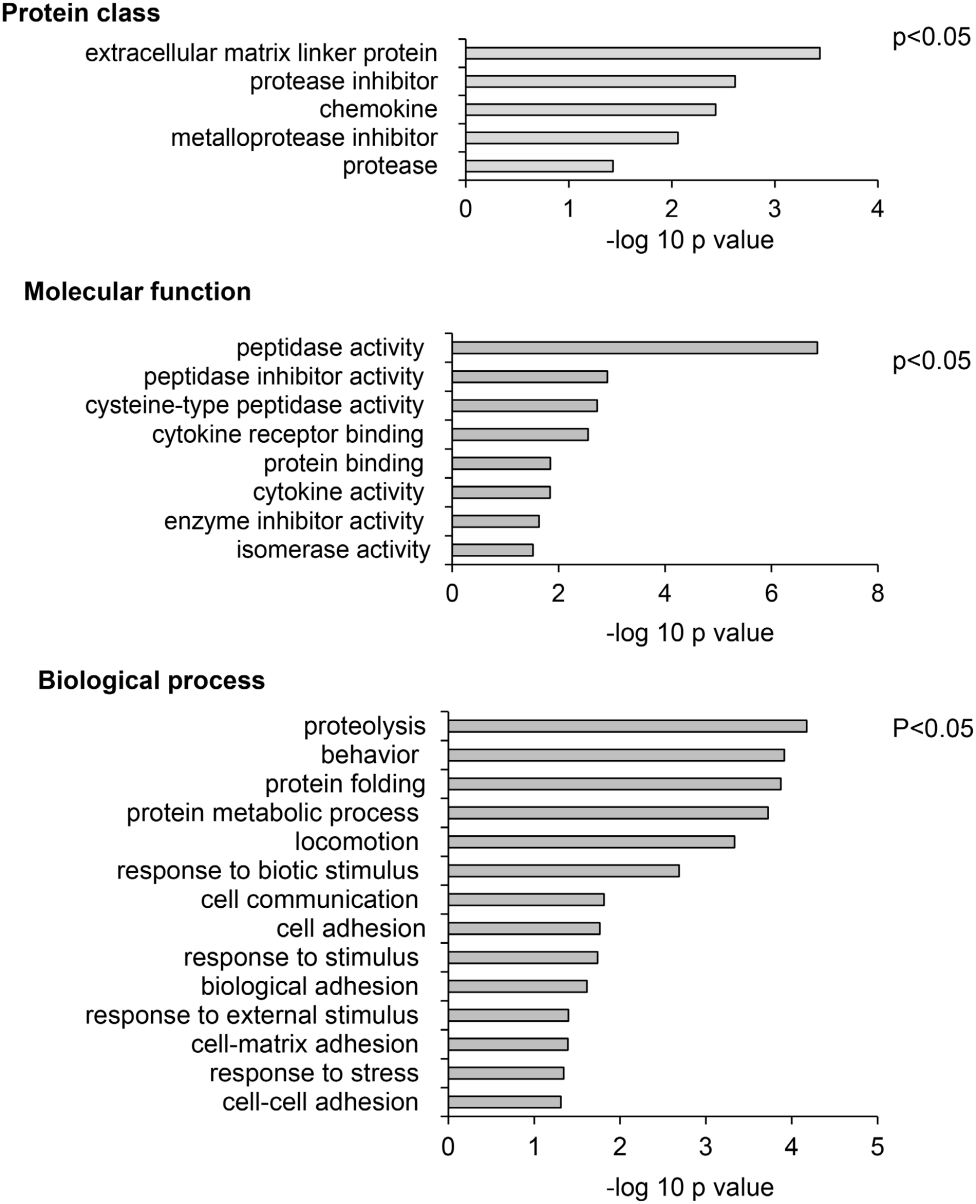
Meta-analysis of SIDLS labeling of the AGS cell secretome identifies proteins involved in inhibition of proteolysis as targets of chemerin Protein ANalysis THrough Evolutionary Relationships (PANTHER) analysis shows, top, enriched protein classes; middle, enriched molecular functions; bottom, enriched biological processes. In each case the –log of the probability is shown and data for p<0.05 included.

Analysis of the list of secretomes using Protein ANalysis THrough Evolutionary Relationships (PANTHER) for significantly enriched (p<0.05) protein classes, molecular functions and biological processes, identified “protease inhibitor” and “metalloprotease inhibitor” as two of the top four of *protein classes* affected. Moreover, “peptidase activity” and “peptidase inhibitor activity” were the top ranked *molecular function* identified, and “proteolysis” was the highest ranked *biological process* identified (Figure 6). Amongst specific targets, decreased expression of TIMP-1 and TIMP-2 were identified as significantly reduced in response to chemerin.

### TIMP-1 and TIMP-2 in AGS media are depressed by chemerin and inhibit migration and invasion

**Figure 7:**
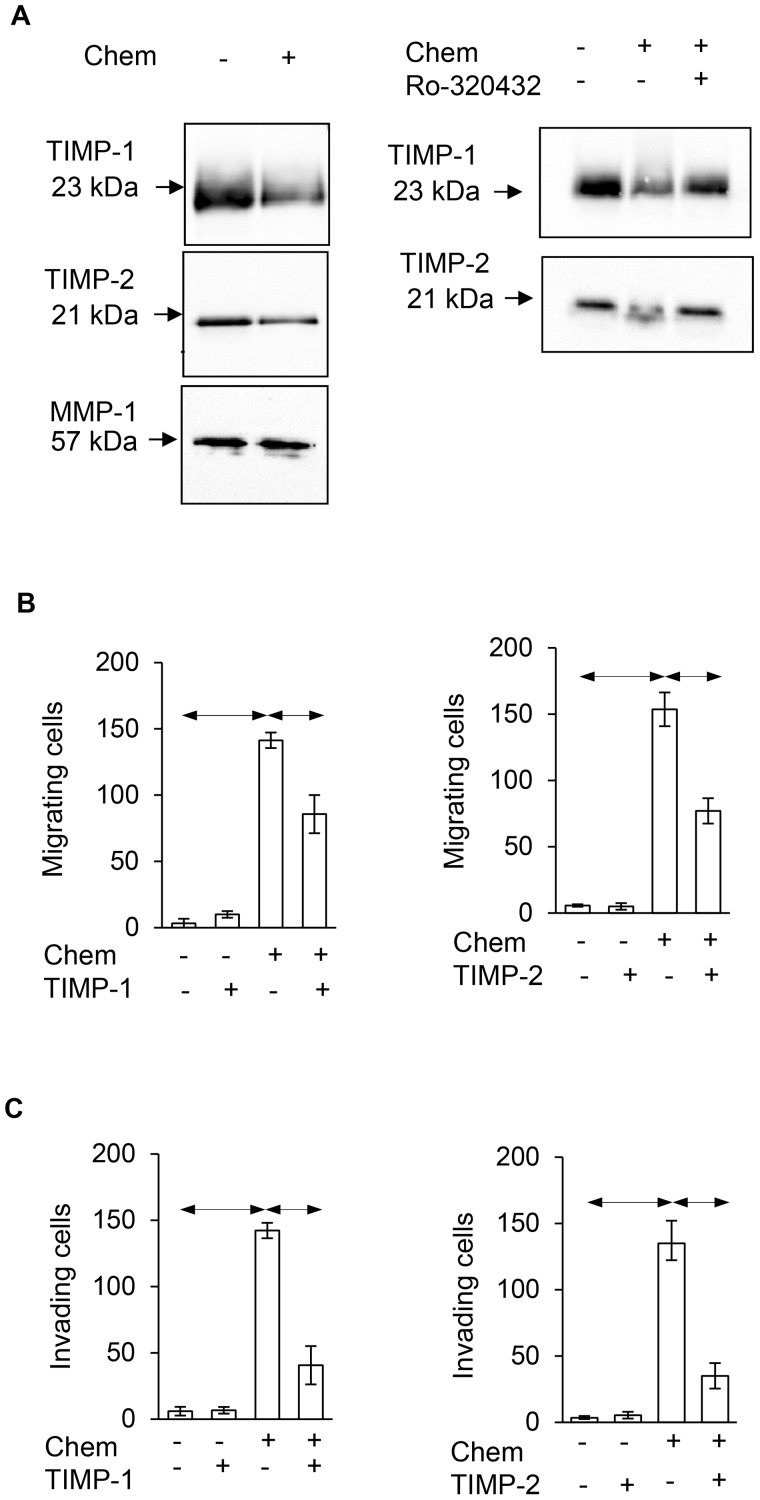
Chemerin stimulates migration and invasion via suppression of TIMP-1 and TIMP-2 **(A)** Left, Western analysis showing chemerin decreases the abundance of TIMP-1 and TIMP-2, but not MMP-1 in AGS cell media; right, the effect of chemerin is inhibited by Ro-320432. **(B)** The effect of chemerin on AGS cell migration is suppressed by addition of TIMP-1 (left; 2.1 nM) and TIMP-2 (right; 2.5 nM). **(C)** Similarly, the effect of chemerin on AGS cell invasion is suppressed by addition of TIMP-1 (left) and TIMP-2 (right). Horizontal bars, p<0.05, ANOVA.

In order to validate the effect of chemerin on TIMP-1 and TIMP-2 we first showed by western blot that in the media of cells treated with chemerin there was depressed TIMP-1 and TIMP-2; in contrast there was no change in the abundance of matrix metalloproteinase (MMP)-1 included for reference (Figure 7A, left). In keeping with the observation that the effects of chemerin are mediated by PKC, the addition of Ro-320432 reversed the decrease in TIMP-1 and TIMP-2 abundance in response to chemerin (Figure 7A, right). In migration experiments, restitution of TIMP-1 and TIMP-2, by addition of exogenous protein, inhibited the response to chemerin (Figure 7B). Moreover, in invasion experiments TIMP-1 and TIMP-2 also inhibited the response to chemerin (Figure 7C).

## DISCUSSION

The chemerin/CMKLR1 system is associated with stimulation of migration and invasion of a wide variety of cells including immune and inflammatory cells, mesenchymal stem cells, vascular smooth muscle and endothelial cells [12, 14, 25, 26]. The role of GPR1 is less well studied. Recently there has been emerging interest in the role of this system in promoting cancer cell migration and invasion [13, 27]. The present data support the idea that gastric adenocarcinoma cells express both CMKLR1 and GPR1, and that both play a role in mediating the effect of chemerin on cancer cell migration and invasion. The actions of chemerin appear to be mediated, at least in part, by PKC-suppression of TIMP-1 and -2 expression.

The role of chemerin in different types of cancer appears to vary. In some cases, chemerin is protective; for example, in melanoma it stimulates recruitment of NK cells suggesting a role in tumor cell immune evasion [28]; and there is evidence of a tumor suppressor function in adrenocortical carcinoma and hepatocellular carcinoma [29, 30]. Set against this, however, increased chemerin expression has been associated with colorectal cancer [31], squamous cell carcinoma of the oral tongue [32] and stomach cancer [20], while the situation in the case of non-small cell lung cancer remains uncertain [33, 34]. The data therefore suggest that depending on the tumor type, chemerin may be associated with either aggressive or protective roles.

The expression in primary tumors of the putative receptors, CMKRL1 and GPR1, has been less well studied than that of chemerin itself. Previously, primary esophageal squamous cancers were shown to express CMKLR1 [13]; neuroblastoma cells also express both receptors and there is evidence that chemerin acts via CMKLR1 in an α-NETA sensitive mechanism to increase MMP-2 to promote tumor growth [35]. We now show that both CMKLR1 and GPR1 are expressed by primary gastric adenocarcinoma cells. Thus in addition, to possible roles of chemerin in modifying the tumor microenvironment by influencing migration of immune and inflammatory cells there is also the potential for direct effects on cancer cell invasion or migration. The balance between the effects of chemerin on migration/invasion of cancer cells on the one hand and immune cells on the other hand, may account for the differences between cancer types as to whether chemerin has a protective or aggressive role.

Two previous studies have noted increased invasion of gastric cancer cells in response to chemerin [20, 21]. By selectively inhibiting expression of each receptor we conclude that both CMKLR1 and GPR1 mediate the effects of chemerin, moreover our findings support a role for PKC as a downstream mediator in both cases. The data therefore add to previous work implicating the RhoA and Rho-associated protein kinase (ROCK) pathway, and other kinases in AGS cell responses to chemerin [21]. AGS cells are relatively well studied and are arguably the cell line of choice for EMT-like responses of gastric adenocarcinoma cells; thus, for example, other well studied gastric cancer cell lines such as MKN45 cells perform poorly in assays of the type used here. It is however, worth noting that we have found that in the case of another upper gastrointestinal adenocarcinoma cell line, namely esophageal OE33 cells, there is a 2-fold increase in migration in response to chemerin in Boyden chamber assays (unpublished observations). It is known that activation of PKC in AGS cells leads to an EMT-like phenotype and in this sense the response of AGS cells to chemerin resembles that to the gastric hormone gastrin which also stimulates migration, invasion, morphological transformation via activation of multiple pathways including PKC [22, 36]. There would now be clear advantages to a systematic study of the mechanism of action of chemerin across a range of gastrointestinal adenocarcinoma cell lines.

Two putative chemerin receptor antagonists, CCX832 [37] and α-NETA [38], have been described. In the present study both were able to produce near complete inhibition of AGS cell responses to chemerin. In vascular cells, CCX832 has been reported to be highly specific for CMKLR1 [39], and GPR1 - although expressed - was considered to be functionally unimportant. Since our siRNA data indicate that both CMKLR1 and GPR1 mediate the effects of chemerin in AGS cells and since both antagonists were capable of inhibiting biological responses it seems likely that both are able to act at the two receptors. For practical purposes it would seem appropriate, therefore, to exercise caution in assigning the actions of these compounds to one receptor or the other.

The elucidation of secretomes is central to an understanding of the dynamics of the tumor microenvironment. Early studies in this area using AGS cells noted changes in plasminogen activator inhibitor (PAI)-2 and PAI-1, and fibroblast growth factor (FGF)2, using microarray or proteomic analyses [40, 41]. More recently, proteomic and microarray analysis of myofibroblast secretomes has identified the importance of MMPs and extracellular matrix proteins [8, 42]. Cell migration and invasion whether of cancer or stromal cells requires dynamic changes in the capacity for extracellular protein digestion which in turn reflects a balance between the release and activation of proteases, particularly members of the MMP family, and their inhibitors such as the TIMPs [43]. One of the novel findings of the present study is that suppression of TIMP-1 and -2 is a downstream response to chemerin mediated by PKC.

Gastric cancer remains a devastating disease associated with high mortality. Early detection favours survival but even so there is a need for new approaches to treat progression of the disease. The present study raises the possibility that the chemerin system may be a useful target for slowing the migration and invasion steps that lead to metastasis. The generation of antagonists for two of the main chemerin receptors (CMKLR1 and GPR1) encourages the idea that it may be feasible to develop relevant new therapeutic approaches for the inhibition of metastasis in these tumors.

## MATERIALS AND METHODS

### Patients

Formalin-fixed, paraffin embedded, surgical resection material from the gastric tumors of 15 patients were used; the characteristics of the patients, including TNM staging [44], have previously been described [8]. All patients gave informed consent and the study was approved by the University of Szeged Ethics Committee.

### Cells

Gastric cancer cells (AGS) were obtained from American type culture collection (VA, USA). Two different gastric CAMs were used that had been generated previously and have been described [8, 11]. AGS cells and myofibroblasts were cultured as previously described [41, 45].

### Immunohistochemistry

Paraffin embedded sections were processed for immunohistochemistry using antigen retrieval and stained with rabbit polyclonal antibodies to CMKLR1 (Millipore, MA, USA) or GPR1 (Abcam, Cambridge, UK) as previously described [13]. Stromal and epithelial compartments were scored separately for staining intensity on a four point scale (0–3) by two independent pathologists and the percentage of stained cells at each intensity recorded. Controls in which first antibody was either omitted or substituted with control rabbit IgG yielded negative results (ie scored as 0).

### Immunocytochemistry

Cells were formalin-fixed (4% w/v), permeabilised with 0.2% Triton X-100 in PBS (PBS-T) for 30 min at room temperature (RT) and processed for immunohistochemistry as previously described [13] using antibodies to CMKLR1 (Millipore) or GPR1 (Abcam) followed by incubation with the appropriate fluorescein secondary antibodies raised in donkey (Jackson Immunoresearch, Soham, UK), and mounted with Vectashield containing DAPI (Vector Laboratories, Peterborough, UK). Slides were viewed using a Zeiss Axioplan-2 microscope (Zeiss Vision, Welwyn Garden City, UK) and images were captured at 40× magnification.

### Conditioned media

Two gastric cancer myofibroblasts (1.5 × 10^6^ cells) were separately plated in T-75 falcon flasks and maintained at 37°C in a 5% v/v CO_2_ atmosphere for 24 h in FM. Cultures were then washed 3 times with sterile PBS and incubated in 15ml serum free (SF) media for 24 h. Conditioned media were collected, centrifuged (7 min, 800 x g, 4°C) and aliquots stored at −880°C until further use.

### Morphological transformation

AGS cells were seeded at a density of 40,000 cells per well in 6 well plates and allowed to form colonies of an average size of about 30 cells; as described previously these conditions yield sub-confluent cultures that are optimal for visualization of morphological changes [22]. Cell were changed to serum free medium and treated with the active C-terminal nonapeptide of chemerin (GeneScipt, Piscataway, NJ, USA), phorbol 12-myristate 13-acetate (PMA; Sigma, Dorset, UK), Ro-320432 (Sigma), CCX832 (ChemoCentryx, Mountain View, CA, USA) or α-NETA (*N*,*N*,*N*-Trimethyl-γ-oxo-1-naphthalenepropanaminium iodide; Sigma), as appropriate. Five fields captured at 0 and 7 h were quantified for the proportion of cells extending processes, expressed as a percentage.

### Cell migration and invasion assays

Scratch wound migration assays were performed on confluent monolayers of AGS cells as previously described [41]. Transwell migration and invasion chemotaxis assays were performed using BD inserts (Corning, NY, USA) as previous described (25,000 cells per insert) [46]. Chemerin or CAM-conditioned medium (CM) were added in the lower well together with CCX832, α-NETA, Ro-320432, human recombinant TIMP-1 or TIMP-2 (R&D Systems), or vehicle, as appropriate.

### CMKLR1 and GPR-1 knockdown

Cells were transiently transfected using Amaxa^™^ Cell line Nucleofector^™^ kits V using the program T-19 for high transfection efficiency (Amaxa, Köln, Germany) according to manufacturer's instructions. AGS cells were treated with scrambled or validated siRNAs (3μM) for CMKLR1 and GPR-1 (Invitrogen, Paisley, UK) [12]. The efficiency of knockdown for CMKLR1 and GPR-1 was verified by immunocytochemistry.

### Proteomic analysis

Putative chemerin targets in the AGS secretome were identified using a stable isotope dynamic labeling of secretomes (SIDLS) approach based on that recently described [23, 24]. Briefly, AGS cells (10^6^ cells, 10cm dishes, approximately 70% confluency) were incubated with chemerin (10 nM), or not, for 24 h; for the last 6 h the medium was changed to one containing either ^13^C_6_ lysine and ^13^C_6_ arginine (heavy label; chemerin), or ^12^C_6_ lysine and ^12^C_6_ arginine (control, light label), to dynamically label secreted proteins. Media were then collected, pooled and centrifuged (800 x g, 7 min). StrataClean resin (Agilent Technologies Ltd., Wokingham, UK) was used to capture proteins in media samples prior to tryptic digestion as previously described [23] and peptide separation using an Ultimate 3000 nano system (Dionex/Thermo Fisher) coupled to a Q-Exactive quadrupole Orbitrap mass spectrometer (Thermo Fisher). SIDLS data were searched and analysed using MaxQuant 1.1.1.36 against the human IPI database v3.68 using the recommended default settings.

### Secreted protein search and GeneOntology analsysis

Data were filtered with a FDR of 1–5% for heavy to light ratio peptides with cut-off 0.01 and imported into Uniprot to generate fasta sequence files. A search for proteins with signal peptides ie classical secretory proteins (D cut off >0.5) was performed using SignaIP v.4.0. The dataset of secreted proteins exhibiting signal peptides was then used in PANTHERv.10 to identify significantly enriched (p<0.05) protein classes, molecular functions and biological processes.

### Western blotting

Medium was concentrated using StrataClean resin (Agilent Technologies Ltd) and processed for western blotting as previously described [13] using antibodies to chemerin (R&D Systems), TIMP-1, TIMP-2 and MMP-1 (R&D Systems).

### Statistics

Results were calculated as mean ± standard error of means (SEM). Student t-test and ANOVA were performed on the data as appropriate with significance at p<0.05 using Systat Software Inc. (London, UK) unless otherwise stated.

## Abbreviations

CAMs: cancer associated myofibroblasts
CM: conditioned media
FDR: false discovery rate
PMA: phorbol 12-myristate 13-acetate, protein kinase C
SIDLS: stable isotope dynamic labeling of secretomes
PKC: protein kinase C
TIMP: tissue inhibitor of matrix metalloproteinase
